# A lack of association between the *IKZF2* rs12619285 polymorphism and coronary heart disease

**DOI:** 10.3892/etm.2015.2282

**Published:** 2015-02-10

**Authors:** HUADAN YE, QINGXIAO HONG, YIRUN LI, XUTING XU, YI HUANG, LIMIN XU, ANNAN ZHOU, YOUPING DENG, SHIWEI DUAN

**Affiliations:** 1 Zhejiang Provincial Key Laboratory of Pathophysiology, School of Medicine, Ningbo University, Ningbo, Zhejiang 315211, P.R. China; 2 Department of Neurosurgery, Ningbo First Hospital, Ningbo, Zhejiang 315010, P.R. China; 3 Rush University Cancer Center, and Department of Internal Medicine and Biochemistry, Rush University Medical Center, Chicago, IL 60612, USA

**Keywords:** *IKZF2*, rs12619285, coronary heart disease, polymorphism

## Abstract

The *IKZF2* rs12619285 polymorphism is associated with the eosinophil count, which has multidimensional functions in the pathogenesis of coronary heart disease (CHD). The aim of the present study was to investigate the contribution of the *IKZF2* rs12619285 polymorphism to the risk of CHD in a Han Chinese population. In total, 721 CHD cases and 631 non-CHD controls were recruited for an association study of the *IKZF2* rs12619285 polymorphism. Genotyping was performed using the melting temperature-shift polymerase chain reaction method. No statistically significant association was observed between the *IKZF2* rs12619285 polymorphism and CHD (odds ratio, 1.139, 95% confidence interval, 0.927–1.334; P=0.17). In addition, subgroup analyses by gender or age were unable to identify any association between *IKZF2* rs12619285 and CHD (P>0.05), and there was no significant correlation between *IKZF2* rs12619285 and the severity of CHD (P>0.05). The power of the case-control study was determined to be 63.3%. In addition, the G allele frequency was calculated as 63.6% in the Han Chinese population, which was similar to the 59.3% recorded for the HapMap Chinese population of Han Chinese individuals living in Beijing, compared with 24.3% in European descendents (HapMap-CEU). Therefore, the results indicated that the *IKZF2* rs12619285 polymorphism was not associated with CHD in a Han Chinese population. The discrepancy in the association between rs12619285 and CHD may be due to the ethnic differences between Han Chinese and European populations.

## Introduction

Coronary heart disease (CHD) is a major cause of mortality and disability worldwide (1). The global number of CHD-induced mortalities is increasing rapidly and is hypothesized to reach 23.3 million by 2030 (2). CHD is induced by the accumulation of plaques on vascular endothelial surfaces (3,4). As a complex disease, CHD is influenced by lifestyle, environmental and genetic factors (1,5). Twin studies have provided evidence that genetic and environmental factors play key roles in the occurrence and development of CHD (6–8).

The *IKZF2* gene is located on chromosome 2q13 and encodes the zinc-finger protein, Helios, which is a member of the Ikaros family of zinc-finger proteins. IKZF2 protein is a functional component in lymphocyte differentiation (9) and plays a key role in the growth of the early hematopoietic system (10). Hematopoietic progenitors can develop into neutrophils, eosinophils, dendritic cells, Langerhans cells, macrophages and osteoclasts (11). Infiltration of eosinophils has been found in the myocardial tissue of patients with hypereosinophilic syndrome (12). The rs12619285 polymorphism of the *IKZF2* gene has been associated with a variation in the blood eosinophil count (13). Furthermore, genes that are involved in the regulation of eosinophil numbers have been shown to be involved in the inflammatory regulation and immune responses that occur during the development of CHD (14–16). Eosinophils, as a type of white blood cell, exert multidimensional functions in the occurrence and development of autoimmune diseases (17), particularly in the pathogenesis of CHD (18,19) by promoting thrombus formation (20).

In addition, a previous study found that the *IKZF2* rs12619285 (G/A) polymorphism in European populations (G allele frequency, 26%; P=5.4×10^−10^) and East Asian populations (G allele frequency, 64%; P=0.017) was significantly associated with CHD (21), although there were large allele differences between the European and East Asian populations. Previously, preliminary results indicated that there was an association between *IKZF2* rs12619285 and CHD in the Chinese Han population [G allele frequency, 62.8%; P=0.07; odds ratio (OR), 1.38, 95% confidence interval (CI), 0.97–1.98], with no departure from the Hardy-Weinberg equilibrium (HWE) in the controls (22). Thus, an association was observed between *IKZF2* rs12619285 and CHD (162 cases and 113 controls); however, the statistical power was only 45.2% (22). The small sample size used in this preliminary study may have been unable to indicate the authentic association between rs12619285 and CHD (22). Thus, the aim of the present study was to investigate the association between the rs12619285 polymorphism of the *IKZF2* gene in CHD patients and non-CHD controls using an increased sample size.

## Materials and methods

### Sample collection

In total, 1,352 samples were collected between September 2011 and July 2013 in Ningbo Lihuili Hospital (Ningbo, China) and Ningbo Yinzhou People’s Hospital (Ningbo, China). CHD cases were confirmed with angiographic evidence that showed vascular stenosis of >50% in at least one major coronary artery. In addition, participants with a history of angioplasty or coronary artery bypass surgeries were classified as CHD cases. Control samples comprised patients whose vascular stenosis was <50% in each of the major coronary arteries (23). The severity of CHD was classified according to the number of major coronary arteries affected by >50% stenosis (single, double and triple). All the participants were unrelated and of Han Chinese origin, habituating in Zhejiang province. Patients were excluded from the study if they suffered from congenital heart disease, cancer and severe liver or kidney diseases. All the blood samples were collected by the same investigator. This study was approved by the Ethical Committees of Ningbo Lihuili Hospital and Ningbo Yinzhou People’s Hospital. All the individuals provided written informed consent.

### Single nucleotide polymorphism genotyping

**Figure 1:**
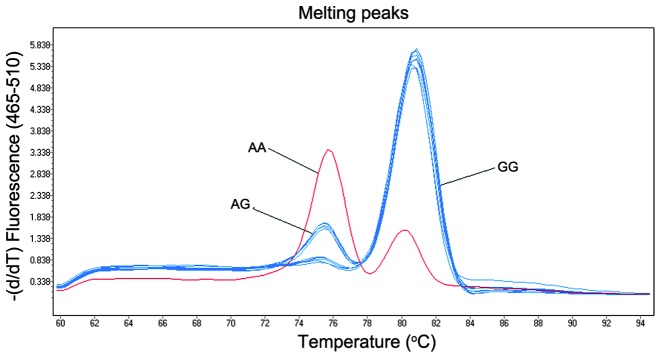
Detection of the three genotypes. The line marked AA indicated homozygous for allele A; the line marked AG indicated heterozygous; and the line marked GG indicated homozygous for allele G.

Genomic DNA from the peripheral blood was extracted using a nucleic acid automatic extractor (Lab-Aid 820; Zeesan Biotech Co., Ltd., Xiamen, China) and all the DNA samples were stored in Tris-EDTA buffer. Genotyping was performed using the melting temperature-shift polymerase chain reaction (PCR) method (24,25). The Tm-shift PCR approach was used to differentiate the two allele-specific PCR products that were amplified using two forward primers and one common reverse primer. The two forward primers comprised one long and one short primer to generate two PCR products with different Tm values. The primers used were as follows: *IKZF2*-g forward, 5′-GCGGGCAGGGCGGCA CCAAGGAAAATGGAGCTTCTG-3′; *IKZF2*-a forward, 5′-GATTACCGACCAAGGAAAATGGAGCTTCTA-3′); and *IKZF2* reverse, 5′-GCCTCTTTAGGTAGGGAAGAG AGAACACA-3′. The PCR amplification program consisted of an initial denaturation at 95°C for 30 sec, followed by denaturation at 95°C for 30 sec, annealing at 59°C for 30 sec and extension at 72°C for 30 sec for 40 cycles, and a final extension at 72°C for 30 sec. PCR was performed using the ABI GeneAmp^®^ PCR System 9700 with the 96-Well Sample Block Module (Applied Biosystems Life Technologies, Foster City, CA, USA). Subsequently, melting curve analysis was performed using a Roche LightCycler 480^®^ fluorescence quantitative PCR instrument (Roche Diagnostics, Basel, Switzerland). The melting curve analysis program was 95°C for 15 sec, 60°C for 30 sec, followed by increasing the temperature by 0.11°C per sec, until a temperature of 95°C was reached. As shown in Fig. 1, the data of the melting curve analysis was obtained by clustering the fluorescence intensity analysis (25).

### Statistical analysis

HWE analysis was performed using Arlequin software (version 3.5; Zoological Institute, University of Bern, Bern, Switzerland) (26). Differences in the genotype and allele frequencies between the case and control groups were calculated using CLUMP 22 software (Institute of Psychiatry, Denmark Hill, London, UK) with 10,000 Monte Carlo simulations (27). OR and 95% CI values were determined using an online program (http://faculty.vassar.edu/lowry/odds2x2.html). The Cochran-Mantel-Haenszel (CMH) test was performed using SAS 9.2 software (SAS Institute, Marlow, UK), while power analysis was conducted using the software of Power and Sample Size Calculation (version 3.0.43; Department of Biostatistics, Vanderbilt University, Nashville, TN, USA) (28). A two-tailed P-value of <0.05 was considered to indicate a statistically significant difference.

## Results

### Distribution of genotypes and alleles in the case and control groups

In total, 721 CHD cases and 631 controls were recruited for the study in order to evaluate the contribution of the *IKZF2* rs12619285 polymorphism to CHD. Genotype distributions of the *IKZF2* rs12619285 polymorphism were shown to not deviate from the HWE in the CHD cases, non-CHD controls and additional subgroups divided by gender or age (P>0.05; Tables I, II and III). The results did not reveal a statistically significant association between the rs12619285 polymorphism and CHD in the case-control study (P=0.17; OR, 1.139, 95% CI, 0.972–1.334; Table I).

### Associations with age and gender

Since the development of CHD may be involved with the interaction between genovariation and the environment (29), the samples were divided into subgroups according to age and gender (30,31), from which subgroup association tests were performed. Subgroup analysis by gender did not identify an association between the rs12619285 polymorphism and CHD in males (P=0.356; OR, 1.099, 95% CI, 0.899–1.344; Table II) or females (P=0.084; OR, 1.269, 95% CI, 0.968–1.663). In addition, an association was not identified between rs12619285 and CHD in the breakdown analysis by age (P>0.05; Table III).

### Associations with genetic models and the severity of CHD

Association tests were also performed using dominant and recessive genetic models. However, no statistically significant difference in the distribution of rs12619285 genotypes or alleles were identified between the case and control subjects (P>0.05; Table IV). In addition, CMH statistical analysis was performed to investigate the association between rs12619285 and the number of arteries with stenosis in the CHD patients. Similarly, no statistically significant association was identified between the rs12619285 polymorphism and the severity of CHD (P>0.05; data not shown). In addition, the G allele frequency was found to be 63.6% in the Han Chinese population, which was similar to the previous preliminary study where the G allele frequency was 62.8% (22). The power of the present case-control study was 63.3%.

## Discussion

In the present case-control study, a statistically significant association between *IKZF2* rs12619285 and CHD was not identified, although there was a borderline statistical difference between the CHD cases and non-CHD controls in the female subgroup (P=0.08). The case-control study included 1,352 individuals that comprised 721 CHD cases and 631 controls. However, the current study was relatively small compared with a previous genome-wide association study that included 12,118 European and 5,212 East Asian individuals (21). Insufficient sample size may explain the negative association observed in the present study (statistical power, 63.3%).

Further analysis indicated that there was an ethnic difference in the allele frequency of the *IKZF2* rs12619285 polymorphism. The G allele frequency of rs12619285 in the HapMap European population was found to be 24.3%, which was much smaller compared with that of the HapMap Han Chinese in Beijing (CHB) population (59.3%). In addition, the G allele frequency was 63.6% in the controls of the present study, which was similar to that of the HapMap-CHB (59.3%) population (32). The discrepancy in the allele frequency may help to explain why the present study failed to confirm the previously identified positive association between the *IKZF2* rs12619285 polymorphism and CHD in the Han Chinese population.

Genetic heterogeneity may be an additional explanation for the negative results. A total of 3,355 polymorphisms have been identified in the *IKZF2* gene. The current study only focused on one polymorphism of the *IKZF2* gene; thus, the function of the *IKZF2* gene may not be fully demonstrated. Future studies should investigate a greater number of polymorphisms in order to improve the understanding of the role of the *IKZF2* gene in the susceptibility of CHD.

IKZF2 protein has been identified at an early phase of development in thymocytes; thus, IKZF2 has been regarded as a key regulator in lymphocyte differentiation (9). In addition, *IKZF2* has been demonstrated to be involved in the earliest hematopoietic differentiation of human embryonic stem cells (33), and neutrophils and eosinophils are developed from hematopoietic progenitors (11). Eosinophils participate in the production and reproduction of inflammation (21), which may promote the development of CHD (34,35). Thus, it was hypothesized that the *IKZF2* gene may play a role in the pathogenesis of CHD.

In conclusion, the present case-control study demonstrated a lack of association between the *IKZF2* rs12619285 polymorphism and CHD in the Han Chinese population. This observation indicated that other *IKZF2* polymorphisms or different genes can affect the variation in blood eosinophil numbers, which may further the understanding into the contribution of inflammatory pathways in CHD.

## Figures and Tables

**Table I:** Distribution of genotypes and alleles in the case and control subjects.

Genotype	Controls (n=631)	Single vessel (n=352)	Double vessels (n=168)	Triple vessels (n=201)	Total cases (n=721)	χ^2^	P-value (df=2)	P-value (df=1)	OR (95% CI)
AA	84	45	11	17	73				
AG	291	156	79	101	336				
GG	256	151	78	83	312	3.546	0.17	0.109	1.139 (0.972–1.334)

OR, odds ratio; CI, confidence interval; df, degree of freedom.

**Table II:** Distribution of genotypes and alleles according to gender.

Gender	Genotype (n)			χ^2^	P-value (df=2)	HWE	Allele (n)		χ^2^	P-value (df=1)	OR (95% CI)
	
	GG	AG	AA				G	A			
Male											
Cases (n=516)	217	242	57			0.441	676	356			
Controls (n=345)	140	157	48	1.588	0.452	0.730	437	253	0.852	0.356	1.099 (0.899–1.344)
Female											
Cases (n=205)	95	94	16			0.326	284	126			
Controls (n=286)	116	134	36	3.534	0.171	0.898	366	206	2.978	0.084	1.269 (0.968–1.663)

OR, odds ratio; CI, confidence interval; df, degree of freedom; HWE, Hardy-Weinberg equilibrium.

**Table III:** Distribution of genotypes and alleles according to age.

	Genotype (n)						Allele (n)				
								
Age (years)	GG	AG	AA	χ^2^	P-value (df=2)	HWE	G	A	χ^2^	P-value (df=1)	OR (95% CI)
≤55											
Cases (n=166)	70	79	17			0.494	219	113			
Controls (n=224)	87	108	29	0.862	0.650	0.667	282	166	0.756	0.385	1.141 (0.848–1.536)
55–65											
Cases (n=244)	99	113	32			1.000	311	177			
Controls (n=243)	95	113	35	0.215	0.898	0.892	303	183	0.200	0.655	1.061 (0.818–1.377)
≥65											
Cases (n=311)	143	144	24			0.148	430	192			
Controls (n=164)	74	70	20	2.654	0.265	0.601	218	110	0.705	0.401	1.130 (0.849–1.503)

OR, odds ratio; CI, confidence interval; df, degree of freedom; HWE, Hardy-Weinberg equilibrium.

**Table IV:** Genotyping under dominant and recessive models.

rs12619285	Dominant (GG/AG+AA)	χ^2^	P-value (df=1)	OR (95% CI)	Recessive (GG+AG/AA)	χ^2^	P-value (df=1)	OR (95% CI)
Total cases	312/409				648/73			
Total controls	256/375	1.009	0.315	1.117 (0.900–1.388)	547/84	3.330	0.068	1.363 (0.977–1.903)
Male cases	217/299				459/57			
Male controls	140/205	0.185	0.667	1.063 (0.806–1.402)	297/48	1.587	0.208	1.301 (0.863–1.963)
Female cases	95/110				189/16			
Female controls	116/170	1.629	0.202	1.266 (0.881–1.818)	250/36	2.884	0.090	1.701 (0.916–3.157)

OR, odds ratio; CI, confidence interval; df, degree of freedom.
